# Irradiation-driven molecular dynamics simulation of the FEBID process for Pt(PF_3_)_4_

**DOI:** 10.3762/bjnano.12.86

**Published:** 2021-10-13

**Authors:** Alexey Prosvetov, Alexey V Verkhovtsev, Gennady Sushko, Andrey V Solov’yov

**Affiliations:** 1 MBN Research Center, Altenhöferallee 3, 60438 Frankfurt am Main, Germany; 2 on leave from Ioffe Physical-Technical Institute, Polytekhnicheskaya 26, 194021 St. Petersburg, Russia

**Keywords:** focused electron beam-induced deposition, irradiation-driven molecular dynamics, irradiation-induced chemistry, platinum nanostructures, reactive force fields

## Abstract

This paper presents a detailed computational protocol for the atomistic simulation of formation and growth of metal-containing nanostructures during focused electron beam-induced deposition (FEBID). The protocol is based upon irradiation-driven molecular dynamics (IDMD), a novel and general methodology for computer simulations of irradiation-driven transformations of complex molecular systems by means of the advanced software packages MBN Explorer and MBN Studio. Atomistic simulations performed following the formulated protocol provide valuable insights into the fundamental mechanisms of electron-induced precursor fragmentation and the related mechanism of nanostructure formation and growth using FEBID, which are essential for the further advancement of FEBID-based nanofabrication. The developed computational methodology is general and applicable to different precursor molecules, substrate types, and irradiation regimes. The methodology can also be adjusted to simulate the nanostructure formation by other nanofabrication techniques using electron beams, such as direct electron beam lithography. In the present study, the methodology is applied to the IDMD simulation of the FEBID of Pt(PF_3_)_4_, a widely studied precursor molecule, on a SiO_2_ surface. The simulations reveal the processes driving the initial phase of nanostructure formation during FEBID, including the nucleation of Pt atoms and the formation of small metal clusters on the surface, followed by their aggregation and the formation of dendritic platinum nanostructures. The analysis of the simulation results provides spatially resolved relative metal content, height, and growth rate of the deposits, which represents valuable reference data for the experimental characterization of the nanostructures grown by FEBID.

## Introduction

The controllable fabrication of nanostructures with nanoscale resolution remains a considerable scientific and technological challenge [1]. To address this challenge, novel techniques exploiting the irradiation of nanosystems with collimated electron and ion beams have been developed [2–3]. One of these techniques is electron beam lithography (EBL), which is similar to conventional optical lithography but relies on the change of solubility after electron exposure of irradiation-sensitive resists. The EBL process includes the surface coating with a resist, exposure to the energetic electron beam, and further development of the surface to remove irradiated or non-irradiated material. Another technique, focused electron beam-induced deposition (FEBID) [2–5], is based on the irradiation of precursor molecules [6] by high-energy electrons while they are being adsorbed upon a substrate. Electron-induced decomposition releases the non-volatile part of the precursor molecules, forming a deposit on the surface, whereas the volatile fragments are pumped out of the working chamber. Nowadays, FEBID permits the fabrication of nanostructures with sizes down to a few nanometers, which is similar to the size of the incident electron beam [7].

To date, FEBID mainly relies on precursor molecules developed for chemical vapor deposition (CVD), a process mainly governed by thermal decomposition, while dissociation mechanisms in FEBID are predominantly electron-induced reactions. While primary electron (PE) energies during FEBID are typically between 1 and 30 keV, chemical dissociation is most efficient for low-energy (up to several tens of electronvolts) secondary electrons (SE) created in large numbers when a high-energy PE beam impinges on a substrate. Secondary electrons are emitted from the substrate and the deposit, substantially complicating the electron-induced chemistry that governs FEBID. As a result, the precise control of size, shape, and chemical composition of the fabricated nanostructures is still a technological challenge [7], mainly originating from the lack of molecular-level understanding of the irradiation-driven chemistry (IDC) underlying formation and growth of nanostructures.

Further advances in FEBID-based nanofabrication require a deeper understanding of the relationship between deposition parameters and physical characteristics of fabricated nanostructures (e.g., size, shape, purity, and crystallinity). Further advancement of the existing experimental techniques and molecular-level computational modeling can provide insights into the fundamental mechanisms of electron-induced precursor fragmentation and the corresponding mechanisms of nanostructure formation and growth using FEBID.

Until recently, most computer simulations of FEBID and nanostructure growth have been performed using a Monte Carlo approach and diffusion–reaction theory [2,8–10], which allow for simulations of the average characteristics of the process concerning local growth rates and the nanostructure composition. However, these approaches do not provide any molecular-level details regarding structure (crystalline, amorphous, or mixed) and the IDC involved. At the atomic level, quantum chemistry methods have been utilized to analyze the adsorption energies and optimized structures of different precursor molecules deposited on surfaces [11–12]. Nevertheless, ab initio methods are only applicable to relatively small molecular systems with a typical size of up to a few hundred atoms. This makes ab initio approaches of limited use to describe the irradiation-induced chemical transformations occurring during the FEBID process.

A breakthrough into the atomistic description of FEBID has been achieved recently by means of irradiation-driven molecular dynamics (IDMD) [13], a novel and general methodology for computer simulations of irradiation-driven transformations of complex molecular systems. This approach overcomes the limitations of previously used computational methods and describes FEBID-based nanostructures at the atomistic level by accounting for quantum and chemical transformation of surface-adsorbed molecular systems under focused electron beam irradiation [13–16].

Within the IDMD framework various quantum processes occurring in an irradiated system (e.g., ionization, bond dissociation via electron attachment, or charge transfer) are treated as random, fast, and local transformations incorporated into the classical MD framework in a stochastic manner with the probabilities elaborated on the basis of quantum mechanics [13].

Major transformations of irradiated molecular systems (e.g., molecular topology changes, redistribution of atomic partial charges, or alteration of interatomic interactions) are simulated by means of MD with reactive force fields [17–18] using the advanced software packages MBN Explorer [19] and MBN Studio [20]. MBN Explorer is a multi-purpose software package for multiscale simulations of structure and dynamics of complex “meso-bio-nano” systems [14]. MBN Studio is a powerful multi-task toolkit enabling to set up and start MBN Explorer calculations, to monitor their progress, to examine calculation results, to visualize inputs and outputs, and to analyze specific characteristics determined by the output of MD simulations [20].

In a pioneering study [13], IDMD was successfully applied for the simulation of FEBID of W(CO)_6_ precursors on a SiO_2_ surface and enabled to predict the morphology, molecular composition, and growth rate of tungsten-based nanostructures emerging on the surface during the FEBID process. The follow-up study [15] introduced a novel multiscale computational methodology that couples Monte Carlo simulations for radiation transport with IDMD for simulating the IDC processes with atomistic resolution. The spatial and energy distributions of secondary and backscattered electrons emitted from a SiO_2_ substrate were used to simulate electron-induced formation and growth of metal nanostructures after deposition of W(CO)_6_ precursors on SiO_2_.

Investigation of the physicochemical phenomena that govern the formation and growth of nanostructures coupled to radiation is a complex multi-parameter problem. Indeed, different precursor molecules, substrate types, irradiation, replenishment and post-processing regimes, and additional molecular species that may facilitate precursor decomposition can be explored to improve the purity of grown deposits and increase the deposition rate. Therefore, it is essential to develop a comprehensive computational protocol for atomistic simulations of electron-induced nanostructure formation during the FEBID process.

This paper outlines a detailed computational methodology for modeling the formation and growth of metal-containing nanostructures during FEBID by means of IDMD, which was developed in previous studies [13,15]. Different computational aspects of the methodology and the key input parameters describing the precursor molecules, the substrate, and the irradiation and replenishment conditions are systematically described. A step-by-step simulation and parameter determination workflow represents a comprehensive computational protocol for simulating and characterizing a broad range of nanostructures created by means of FEBID. The presented methodology has been developed with a focus on the FEBID process using a pulsed electron beam. However, it can be adjusted and extended by including processes relevant to different EBL or FEBID regimes, for example, variation of the replenishment rates, concentration, and distribution of molecules added and removed from the system.

The formulated computation protocol is applied to simulate the FEBID of Pt(PF_3_)_4_, a widely studied precursor molecule [21–26], on a fully hydroxylated SiO_2_ surface. As such, this work extends the earlier IDMD-based studies [13,15] of the FEBID of W(CO)_6_ towards another precursor molecule that has been commonly used to fabricate platinum-containing nanostructures. In contrast to the earlier studies [13,15] we consider the case of low precursor surface coverage (below one monolayer), in which surface diffusion plays an important role in the formation of deposits. In particular, we focus on the atomistic characterization of the initial stage of the FEBID process, including nucleation of Pt atoms, formation of small metal clusters on the surface followed by their aggregation, and eventually, the formation of dendritic platinum nanostructures.

The morphology of the nanostructures grown during the FEBID process has not yet been thoroughly investigated on the atomistic level although it governs many physical properties such as electrical and thermal conductivity, and magnetic properties [27–28]. Atomistic simulations provide insights into the internal structure of the deposits and its evolution depending on the regimes of the FEBID process. In this study, morphology, size, and metal content of the deposited Pt-containing material are quantitatively analyzed as functions of electron fluence and adsorbate concentration.

## Computational Methodology

Computer simulations of the FEBID process of Pt(PF_3_)_4_ have been performed by means of the MBN Explorer software package [19]. The MBN Studio toolkit [20] has been utilized to create the systems, prepare all necessary input files and analyze simulation outputs.

FEBID operates through successive cycles of precursor molecule replenishment on a substrate and irradiation by a tightly focused pulsed electron beam, which induces the release of metal-free ligands and the growth of metal-enriched deposits. It involves a complex interplay of phenomena taking place on different temporal and spatial scales: (i) deposition, diffusion, and desorption of precursor molecules on the substrate; (ii) transport of the primary, secondary, and backscattered electrons; (iii) electron-induced dissociation of the adsorbed molecules; and (iv) the follow-up chemistry. Each of these phenomena requires dedicated computational and theoretical approaches.

**Figure 1:**
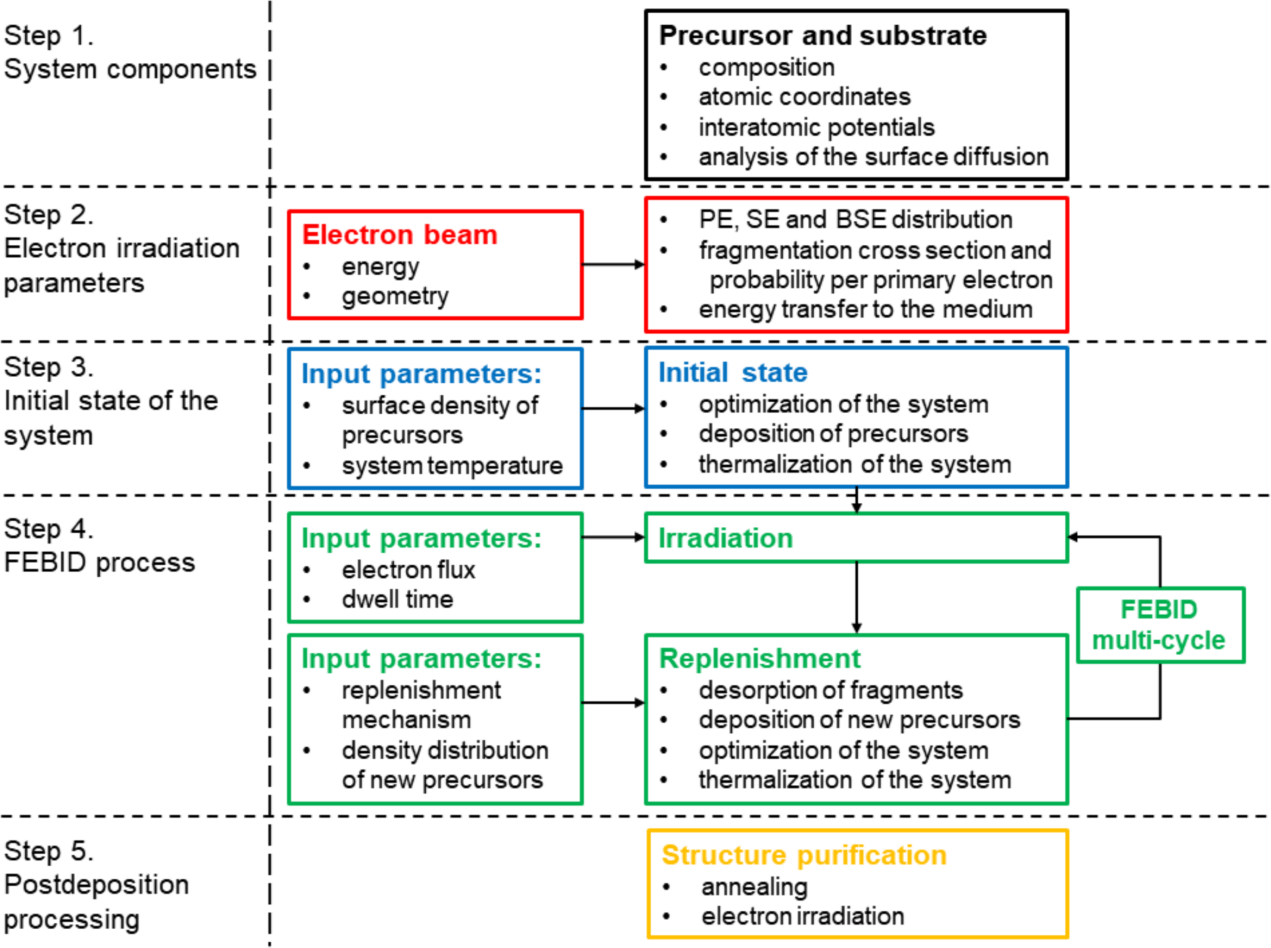
Atomistic IDMD-based simulation protocol of the FEBID process by means of MBN Explorer [19] and MBN Studio [20] software packages.

The general workflow of the atomistic IDMD-based simulation of the FEBID process and the corresponding input parameters are summarized in Figure 1. The formulated procedure for the atomistic simulation of FEBID is general and applicable to any combination of precursor, substrate, and electron beam with minimum variations of the case-specific parameters. The methodology can also be adjusted to simulate the nanostructure formation by other nanofabrication techniques using electron beams, such as direct-write EBL [3].

In the following section, all steps of the protocol shown in Figure 1 are described in detail, going from the system specification (step 1) to the multi-cycle simulation of the FEBID process (step 4) and the simulation of post-deposition processing (step 5). In step 1, one needs to choose the type of precursor molecules and the substrate, specify atomic coordinates and the parameters of interatomic potentials. In step 2, the spatial and energy distributions of the electron irradiation field, the fragmentation cross section, as well as the energy deposited into the system during the fragmentation process are specified. The initial state of the adsorbed molecules to be exposed to electron-beam irradiation is created in step 3. This follows by the multiple cycling of irradiation and replenishment phases in step 4 using the experiment-related input parameters. Additional post-growth processing that enables purification of the deposited structures is simulated in step 5. All these steps are described in greater detail below.

### Step 1. System components: precursor and substrate

The first step of the FEBID simulation procedure shown in Figure 1 concerns the specification of a precursor molecule and a substrate. The selection of the system components is usually linked to available experiments. Common experimentally used substrate materials are, for example, SiO_2_, Si, Au, and amorphous carbon. A choice of the substrate affects the adsorption, desorption, and diffusion of precursor molecules on the surface as well as the yields of secondary and backscattered electrons. These quantities affect the fragmentation rate of the adsorbed precursor molecules and, hence, the nanostructure growth rate.

The chemical composition and geometry of both the precursor and the substrate are specified using the standard .pdb or .xyz file formats. Atomic coordinates for many different precursor molecules can be found in online databases, for example, the NIST Chemistry WebBook (https://webbook.nist.gov/) and PubChem (https://pubchem.ncbi.nlm.nih.gov/), or can be determined via DFT calculations [11–12,29].

MBN Explorer and MBN Studio enable the creation of various crystalline substrates for which the unit cell and translation vectors are specified. The software tools enable also creating amorphous substrates, for example, amorphous silica or amorphous carbon, which are commonly used in FEBID and surface science experiments.

The structure of precursor molecules, their interaction with a substrate, and the dynamics of nanostructure formation and growth are influenced by interatomic interactions among the constituents of the system.

#### Interatomic potentials

The precursor molecules are described via the reactive CHARMM (rCHARMM) force field [17–18]. rCHARMM permits simulations of systems with dynamically changing molecular topologies, which is essential for modeling the precursor fragmentation and the formation of metal-containing nanostructures. A detailed description of rCHARMM is given in [17], while its key aspects are summarized below.

The covalent bond interactions are described in rCHARMM by means of the Morse potential:

[1] $$U^{\text{bond}}\left(r_{ij}\right)=D_{ij}\left[e^{-2\beta_{ij}\left(r_{ij}-r_{0}\right)}-2e^{-\beta_{ij}\left(r_{ij}-r_{0}\right)}\right].$$

Here *D_ij_* is the dissociation energy of the bond between atoms *i* and *j*, *r*_0_ is the equilibrium bond length, and the parameter


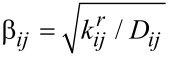


(where 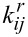 is the bond force constant) determines the steepness of the potential. The bonded interactions are truncated at a user-defined cutoff distance that characterizes the distance beyond which the bond is considered broken and the molecular topology of the system changes. The bond energy given by Equation 1 asymptotically approaches zero at large interatomic distances.

The rupture of covalent bonds in the course of simulation employs the following reactive potential for the valence angles [17]:

[2] $$U^{\text{angle}}\left(\theta_{ijk}\right)=2k_{ijk}^{\theta }\sigma \left(r_{ij}\right)\sigma \left(r_{jk}\right)\left[1-\cos\left(\theta_{ijk}-\theta_{0}\right)\right],$$

where θ_0_ is the equilibrium angle, *k*^θ^ is the angle force constant, and the sigmoid function

[3]
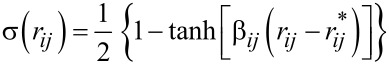


describes the effect of bond breakage. Here,


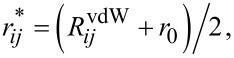


with *r*_0_ being the equilibrium distance between two atoms involved in the angular interaction and 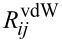 being the van der Waals radius for those atoms.

In the case study considered, the initial geometry of a Pt(PF_3_)_4_ molecule is determined via DFT calculations and then optimized using MBN Explorer. The rCHARMM parameters for a Pt(PF_3_)_4_ molecule are determined from a series of DFT-based potential energy scans, similar to how it was done in [29] for a W(CO)_6_ precursor molecule. In brief, the DFT calculations are performed using the Gaussian 09 software [30] employing the B3LYP exchange–correlation functional and a mixed LanL2DZ/6-31+G(d,p) basis set, wherein the former set describes the Pt atom and the latter is applied to P and F atoms. The geometry of the molecule is optimized first (states with spin multiplicity *M* = 1, 3, and 5 were considered) and a potential energy scan is then performed for Pt–P and P–F bonds to calculate equilibrium bond lengths, dissociation energies, and force constants. The parameters of the bonded and angular interactions for Pt(PF_3_)_4_ are listed in Table 1. In contrast to the previous IDMD-based simulations [13,15] of FEBID of W(CO)_6_, angular interactions are explicitly included in the simulations reported here as they are crucial for describing correctly the tetrahedral structure of PF_3_ ligands bound to the Pt atom.

**Table 1:** Parameters of the covalent bonds and angular interactions for the Pt(PF_3_)_4_ molecule employed in the present simulations.

bond type	Pt–P	P–F	angle type	P–Pt–P	Pt–P–F	F–P–F

*r*_0_ (Å)	2.30	1.59	θ_0_ (°)	109.5	119.3	98.2
*D_ij_* (kcal/mol)	31.1	135.2				
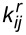 (kcal·mol^−1^·Å^−2^)	120.0	120.0	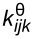 (kcal·mol^−1^·rad^−2^)	76.4	28.0	100.0

Following the earlier studies of FEBID by means of IDMD [13,15], the fully hydroxylated SiO_2_ substrate is fixed in space in the course of simulations to speed up the simulations. The interaction between the ideal SiO_2_-H surface and adsorbed Pt-containing precursor molecules and fragments is governed by weak van der Waals bonding, which agrees with the results of [31]. The van der Waals forces between the atoms of the substrate and the adsorbed molecules are described by means of the Lennard-Jones potential:

[4] $$U_{\text{LJ}}\left(r_{ij}\right)=\varepsilon_{ij}\left[{\left(\frac{r^{\text{min}}}{r_{ij}}\right)}^{12}-2{\left(\frac{r^{\text{min}}}{r_{ij}}\right)}^{6}\right],$$

where 
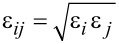
 and 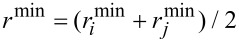. Note that partial hydroxylation, surface defects, and broken O–H bonds may lead to a stronger interaction between Pt and substrate atoms, causing agglomeration of Pt atoms at specific sites. In this case, the covalent interaction between Pt atoms and atoms of the SiO_2_ can be described using the well-established interatomic potentials [31]. These effects can be addressed by the presented methodology in future studies.

**Figure 2:**
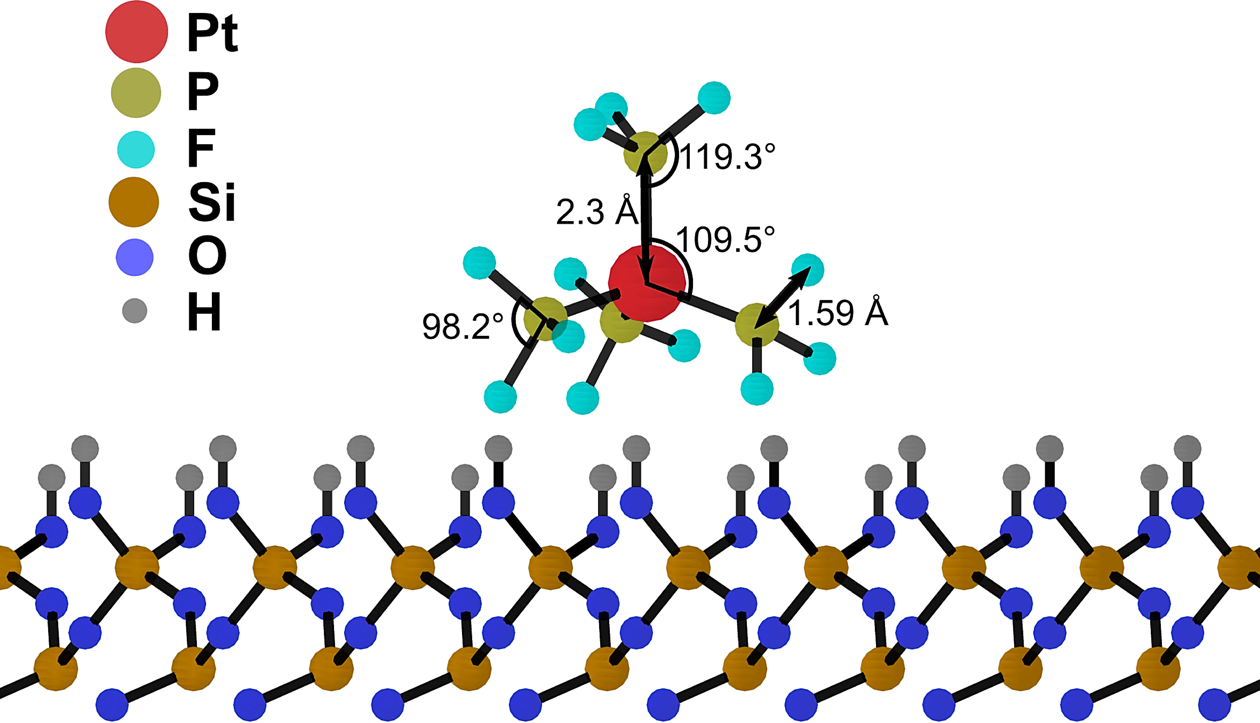
Side view of an optimized single Pt(PF_3_)_4_ molecule adsorbed on a SiO_2_-H substrate. The energy minimization calculation is performed using the interatomic potentials from Equations 1–4.

The van der Waals parameters for all atoms in the system are taken from [32–34] and are summarized in Table 2. The parameters of the van der Waals interaction have been verified by simulating the surface diffusion of a single Pt(PF_3_)_4_ molecule on top of a fully hydroxylated SiO_2_ layer. Although the surface diffusion data for Pt(PF_3_)_4_ on hydroxylated or pristine SiO_2_ are not available, the obtained value of the surface diffusion coefficient, *D* = 2.85 × 10^−7^ cm^2^/s at 300 K, is within the range of values known for the typical surface diffusion coefficient of FEBID precursors [6–7,13]. Figure 2 shows the optimized geometry of a Pt(PF_3_)_4_ molecule adsorbed on a SiO_2_-H substrate and indicates the values of equilibrium covalent bond lengths and angles.

**Table 2:** Parameters of the Lennard-Jones potential describing the van der Waals interaction. ε is the depth of the potential energy well and *r*^min^ is the interatomic distance corresponding to the potential energy minimum.

atom	ε (kcal/mol)	*r*^min^/2 (Å)	ref.

Pt	15.72	1.43	[32]
P	0.32	2.08	[33]
F	0.07	1.74	[33]
Si	0.30	1.60	[34]
O	0.26	1.76	[34]
H	0.02	1.00	[34]

The interaction between metal atoms in the nanostructures formed on a surface can be described via common many-body potentials of the embedded-atom-method type, for example, Sutton–Chen [35] or Gupta [36] potentials. In this study, the interaction between Pt atoms in the formed clusters is described by means of the Gupta potential [36] with a cutoff distance of 7 Å.

Atoms of both the precursor molecules and the substrate may carry non-zero partial charges, and thus the electrostatic interaction should also be taken into consideration. However, accounting for this interaction slows down the simulations significantly. In the present study, test simulations of the Pt(PF_3_)_4_ adsorption and diffusion on SiO_2_-H with non-zero and zero partial charges on the Pt(PF_3_)_4_ molecules have been performed. On the basis of the results obtained, it was concluded that accounting for the Coulomb interaction makes a negligible difference in the structure of adsorbed molecules and their dynamics on the surface. Thus, in the following FEBID simulations the Coulomb interaction is omitted in order to improve the computational performance.

### Step 2. Electron irradiation parameters

Parameters of the electron interaction with a substrate and precursor molecules should be determined in step 2 of the protocol shown in Figure 1. First, one needs to determine the spatial distribution of secondary (SE) and backscattered (BSE) electrons produced due to the collision of the PE beam with the substrate. The convolution of the SE and BSE flux density with the fragmentation cross section of the precursor molecule determines the fragmentation probability of precursors at any space point within the system per primary electron. The electronic collisions with precursor molecules lead to their electronic excitation followed by the fragmentation and energy transfer to the recoil fragments. In the following subsections these processes are discussed in detail.

#### PE, SE, and BSE distributions

The energy and the shape of the PE beam are used to define the distributions of SE and BSE. Typical PE energies used in the FEBID process vary from 1 to 30 keV [5]. One can simulate a tightly focused electron beam with different geometries (e.g., a cylindrical beam or a uniform broad beam covering the whole simulated surface). The latter case is relevant for surface science experiments on irradiation of thin films of adsorbed precursor molecules.

The yields of SE and BSE and the corresponding spatial distributions depend on the energy of the PE beam and the material of a substrate. These distributions can be obtained by means of Monte Carlo (MC) simulations of electron transport [37]. There are several codes suitable for this purpose with different possibilities and limitations, for example, SEED [38], Geant4 [39], and PENELOPE [40]. The yield of SE generated in various materials can also be evaluated by means of analytical models [41].

The obtained spatial and energetic distributions of PE, SE, and BSE are tabulated. The spatial distribution of electrons is defined on a cubic grid covering the whole simulation box; the grid consists of voxels with the size of 1 nm. The energy distribution of electrons is tabulated with a step of 1 eV. In order to permit a straightforward adjustment of the simulated electron flux, the electron distributions are scaled to a single primary electron per unit area within the irradiated surface area. At a small thickness of the deposited material, when the contribution of the deposit to the yield and distribution of electrons is negligible and the fragmentation process occurs mainly in the vicinity of the substrate surface, the radiation field can be defined once as a function of only the surface coordinates. In general, the electron distributions can be recalculated in the course of the FEBID process to account for electron interactions within the deposits. This adjustment is important for simulating the growth of three-dimensional (3D) structures.

**Figure 3:**
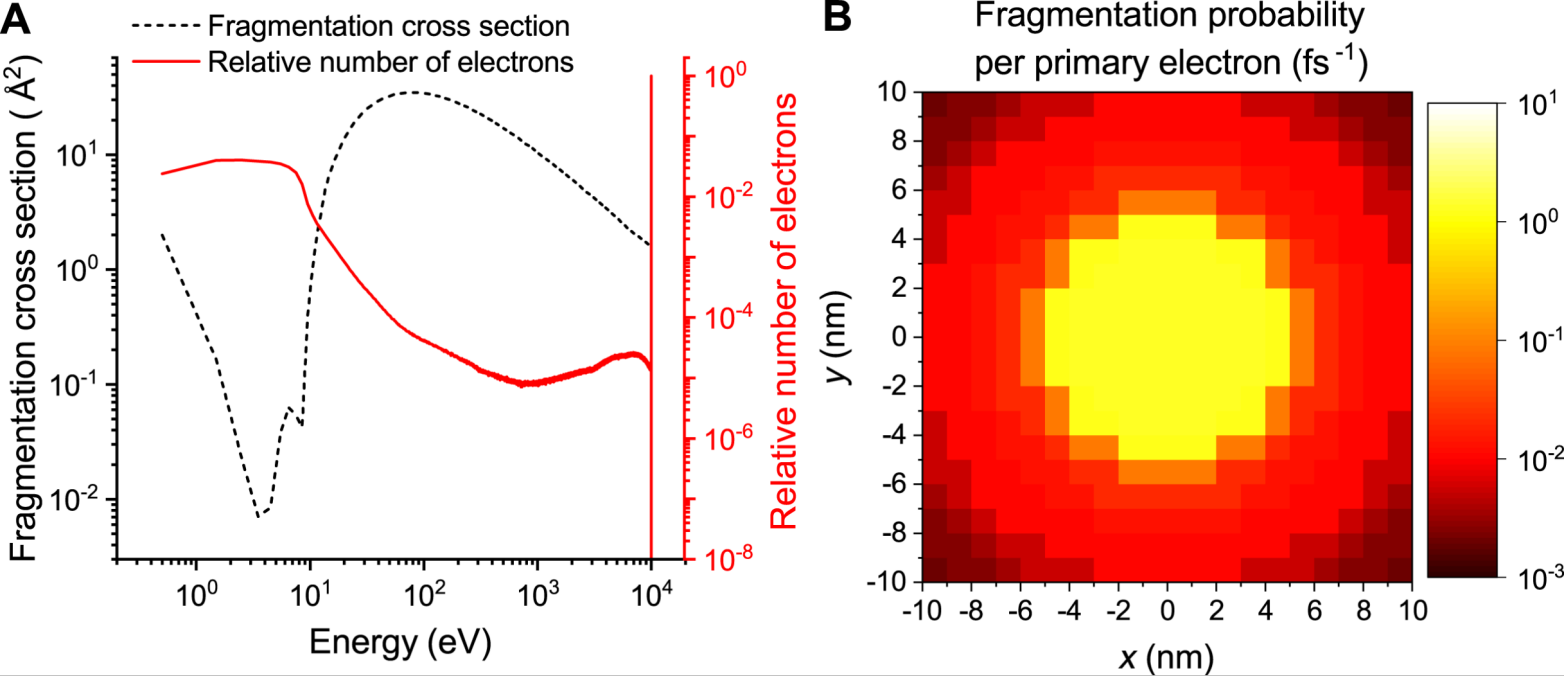
(A) Total electron impact fragmentation cross section of Pt(PF_3_)_4_ including DEA and DI contributions (dashed line) and the relative number of electrons of specific energy per PE [15] including SE and BSE (solid line). (B) Spatial distribution of the fragmentation probability of Pt(PF_3_)_4_ on SiO_2_-H, irradiated with a 10 keV electron beam focused within the beam spot of 5 nm radius. The presented distribution is based on the defined PE flux of 1 Å^−2^·fs^−1^.

In this study we employ the distribution of electrons calculated previously [15] using the SEED code and consider a cylindrical PE beam with a radius of 5 nm and energy of 10 keV. The number of generated electrons (that is the sum of SE and BSE contributions) of specific energy per primary electron [15] is shown in Figure 3A by the solid red line. The dashed line shows the Pt(PF_3_)_4_ fragmentation cross section, discussed further in the text. Figure 3B shows a spatial distribution of the fragmentation probability of Pt(PF_3_)_4_ per PE, calculated using the electron distributions and the Pt(PF_3_)_4_ fragmentation cross section.

#### Fragmentation cross section

An overview of the electron interactions with precursor molecules including possible mechanisms of irradiation-induced molecular fragmentation can be found, for example, in [42]. The main mechanisms of precursor fragmentation are dissociative electron attachment (DEA) at low electron energies below the ionization potential of the molecule and dissociative ionization (DI) at higher electron energies. Neutral dissociation (ND) is another possible fragmentation channel [26], but experimental data on ND are scarce. Accounting for the ND fragmentation channel might lead to an increase of the fragmentation probability. This means that a given fragmentation probability will be achieved at slightly lower beam current. Such a behavior makes the FEBID outcomes not very sensitive to specific fragmentation channels and permits one to compare the characteristics of the simulated FEBID process with experimentally observed characteristics under given experimental conditions.

Electron impact-induced fragmentation experiments performed for a number of precursor molecules revealed [43–45] that the sum of partial cross sections of ionization resulting in the emission of positive ion fragments exceeds significantly (by an order of magnitude) the cross section of ionization without fragmentation of the molecule. On this basis, one concludes that almost every ionizing collision leads to fragmentation; hence, the DI cross section can be approximated by the total ionization cross section. The latter can be calculated by means of the dielectric formalism [46] as it was done in [15] or by means of well-established semi-empirical methods. One of these methods is based on the additivity rule [47], according to which the total molecular ionization cross section is approximated by the sum of ionization cross sections of the constituent atoms or smaller molecular fragments. This method was later improved by taking into account the molecular bonding [48]. The ionization cross section can also be calculated using the Deutsch and Märk (DM) formalism [49] or the binary encounter Bethe (BEB) model [50]. It should be noted that the mentioned methods yield the total electron ionization cross section of the target molecules, that is, the sum of the partial cross sections for all the channels leading to the formation of any charged molecule or fragment [51].

The IDMD approach permits considering several different fragmentation channels. The total fragmentation cross section of a bond is summed up from partial cross sections of DEA and DI channels leading to bond cleavage between atoms. The energy-resolved fragmentation cross section for a specific bond is used to calculate the spatially resolved fragmentation probability *P*(*x*,*y*) per unit time [15]:

[5] $$\begin{array}{c} P\left(x,y\right)=\sigma_{\text{frag}}\left(E_{0}\right)J_{\text{PE}}\left(x,y,E_{0}\right)\\ +\sum_{i}\sigma_{\text{frag}}\left(E_{i}\right)J_{\text{SE/BSE}}\left(x,y,E_{i}\right). \end{array}$$

Here *E_i_* *< E*_0_ is the electron energy discretized in steps of 1 eV, and *J*_PE/SE/BSE_ is the flux density of PE, SE, and BSE, respectively.

The dependence of the fragmentation cross section of Pt(PF_3_)_4_ on the electron energy, σ_frag_(*E*), is shown in Figure 3A by the dashed black line. Here, the dissociation of Pt(PF_3_)_4_ is considered according to the reaction 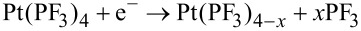. The electron-induced dissociation of P–F bonds has not been considered in the present simulations. The absolute cross section of DEA for Pt(PF_3_)_4_ is taken from the experiment [25]. The DI cross section is approximated from the total ionization cross section of the structurally similar molecule cisplatin (Pt(NH_3_)_2_Cl_2_) [51] by scaling the total number of electrons in the ligands. The accuracy of the cross section evaluation does not affect the results of FEBID simulations as a slight variation of the cross section (e.g., determined by means of more accurate theoretical or computational approaches) would correspond to slightly different values of the beam current.

The product of space- and energy-resolved electron distribution and the precursor fragmentation cross section gives the fragmentation probability per PE per unit time. The calculated probability based on the defined PE flux of 1 Å^−2^·fs^−1^ in the tabulated form for a 20 nm × 20 nm grid covering the simulation box (see Figure 3B) is used as input for the simulations of the irradiation phase of the FEBID process (see step 4).

#### Energy transfer to the medium

A projectile electron interacting with the precursor molecule transfers some amount of energy to the system leaving the molecule in an excited electronic state. In the case of ionization, a fraction of the deposited energy is spent in overcoming the ionization threshold; another fraction is carried away by the ejected electron, while the remaining part is stored in the target in the form of electronic excitations. The latter can involve different molecular orbitals, being of either bonding or antibonding nature. An excitation involving an antibonding molecular orbital evolves through cleavage of a particular bond on the femtosecond timescale, and the excess energy is transferred to the kinetic energy of the produced fragments.

The amount of energy transferred by the incident radiation to the system can be evaluated from quantum mechanical calculations of the processes of energy deposition and excitation [26]. Experimentally, the transferred energy can be estimated based on the measured electron energy loss spectra for a specific precursor molecule [25].

The amount of energy deposited locally into a specific covalent bond of the target and converted into kinetic energy of the two atoms forming the bond is an input parameter for the simulations of FEBID, which may influence the rate of precursor molecule fragmentation. The chosen value of the deposited energy can be verified by comparing the dependence of surface coverage of precursor elements on the electron dose with the experimental data measured by means of X-ray photoelectron spectroscopy (XPS).

Electron energy loss spectra for Pt(PF_3_)_4_ molecules in the gas phase were measured experimentally and compared with time-dependent DFT (TDDFT) calculations in [26]. In the simulations presented below two values of the energy deposited into the molecule, namely 205 kcal/mol (8.9 eV) and 300 kcal/mol (13 eV) were selected. These values correspond to positions of the peaks in the experimentally measured electron energy loss spectra for Pt(PF_3_)_4_ [26].

### Step 3. Initial state of the system

In FEBID experiments precursor molecules are delivered through the gas injection system and fill the scanning electron microscope chamber. Depending on the experimental conditions (temperature, precursor gas pressure, geometry, and position of the gas inlet), precursor molecules adsorb on the substrate surface and form a thin (from sub-monolayer to several monolayer thickness) film.

An explicit evaluation of adsorption and desorption rates for different substrates, temperatures, and concentrations of the adsorbates can be performed by means of MD simulations. It should be stressed that, while the adsorption process can be simulated in detail on the atomistic level, the exact mechanism of precursor deposition does not affect the evolution of the system during irradiation. This means that only the final state of the molecular system, created before the irradiation, is essential for the IDMD simulations.

The outcome of atomistic simulations of precursor adsorption and desorption, that is, the once defined regime, can be used for every related FEBID case study. Due to the complexity and multiple facets of the presented methodology, this question cannot be addressed in the present study and will be systematically explored in follow-up studies.

In step 3 of the computational protocol (see Figure 1) a layer of precursor molecules of specific thickness and density is first created by means of the modeller plugin of MBN Studio [20] and then delivered to the substrate.

The molecular layer is created using the information on the geometry and topology of a single precursor molecule as follows. Precursor molecules are randomly distributed within a box positioned several nm above the substrate. The size of the box is defined according to the specified volume density of the molecules. The atomic coordinates and topology for all molecules in the created precursor layer are saved in .pdb and .psf formats, respectively. Size and molecular density of the precursor layer can be varied to adjust coverage of the substrate surface with precursor molecules corresponding to the experimental conditions.

In order to avoid non-physical overlapping of the atoms, the layer is optimized first using the velocity quenching algorithm for 20,000 steps with a velocity quenching time step of 0.5 fs. After that, the precursor molecules are pulled down to the surface and thermalized at the specified temperature to reach thermal equilibrium. In the simulations the substrate atoms are frozen while all the atoms in precursor molecules are freely moving in space.

In this study a sub-monolayer of Pt(PF_3_)_4_ with the size of 20 nm × 20 nm is optimized, adsorbed on a SiO_2_-H substrate and thermalized at 300 K for 0.1 ns using the Langevin thermostat with a damping time of 0.2 ps. The constructed layer consists of approximately 370 molecules that corresponds to a surface density of 0.9 molecules per nm^2^. These and all the subsequent simulations described further are performed using the Verlet integration algorithm with a time step of 1 fs and reflective boundary conditions. Interatomic interactions are treated using the linked cell algorithm [19] with a cell size of 10 Å.

### Step 4. FEBID process

The FEBID process (step 4 in Figure 1) consists of two phases, namely irradiation and replenishment of the precursors, which are repeated multiple times. The outcome of the FEBID process is governed by a balance of several processes, such as adsorption, desorption, diffusion, and dissociation of molecules. The cumulative contribution of these processes defines the regime in which the FEBID is carried out, that is, the time-dependent concentration and distribution of precursors. Our approach is to replicate the regime representing the system’s state before, during, and after irradiation, without necessarily explicitly simulating all the involved processes. The present methodology allows for the inclusion of additional processes, such as electron-stimulated desorption and chemical reaction between fragments, deposits, and the substrate. Investigating the role of these processes under specific conditions might be a topic for a separate investigation, which goes beyond the scope of the present study.

#### Irradiation

In experiments the irradiation phase of the FEBID process can last for a period (called dwell time τ_d_) from sub-microseconds to sub-milliseconds [7]. During the irradiation phase, the dissociation of the metal–ligand bonds in the precursor molecules occurs resulting in the emergence of reactive molecular fragments with dangling bonds. The dynamics and interaction of the fragments lead to the formation of new bonds between Pt atoms and their coalescence into metal-enriched clusters and bigger structures. As the typical dwell time values are relatively short, adsorption and desorption of precursor molecules within the irradiation phase are assumed negligible.

In this step, the electron-induced precursor fragmentation is simulated by means of IDMD [13] using space-dependent bond dissociation rates for molecules on the substrate. In brief, these rates depend, under steady-state conditions, on (i) the number and energy of electrons crossing the substrate surface at each point per unit time and unit area, which in turn are determined by the PE beam energy *E*_0_ and flux *J*_0_, and (ii) the energy-dependent molecular fragmentation cross section σ_frag_(*E*).

As described above, in step 2 the tabulated spatially resolved fragmentation probability per primary electron is used in the IDMD simulation of the irradiation phase to link the bond dissociation rate to the electron flux. As the realistic experimental time scale for τ_d_ is challenging for all-atom MD, the simulated PE fluxes *J*_0_ (and hence PE beam currents *I*_0_) are rescaled to match the number of PE per unit area and per dwell time as in experiments. The correspondence of simulated results to experimental ones is established through the correspondence of the electron fluence per dwell time per unit area in simulations and experiments [13]. Such an approach is valid in the case when different fragmentation events occur independently and do not induce a collective effect within the system. In this case the irradiation conditions for the adsorbed precursor molecules are the same in simulations and in experiments. This correspondence condition gives

[6] $$I_{\text{exp}}=I_{\text{sim}}\frac{S_{\text{exp}}}{\lambda S_{\text{sim}}}=I_{\text{sim}}\frac{R_{\text{exp}}^{2}}{\lambda R_{\text{sim}}^{2}},$$

[7] $$\lambda =\frac{\tau_{\text{d}}^{\text{exp}}}{\tau_{\text{d}}^{\text{sim}}},$$

where *S*_exp_ and *S*_sim_ are the electron beam cross sections used in experiments and simulations, respectively; *R*_exp_ and *R*_sim_ are the corresponding beam spot radii.

The following experimental irradiation parameters [22] have been used in the simulations performed in the present study: electron current *I*_exp_ = 2.8 nA and estimated beam spot radius *R*_exp_ = 40 nm. Although in the experiments [22] irradiation was performed with a continuous electron beam, we consider the dwell time of a single irradiation cycle to be 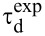 = 1 ms. This corresponds to the PE flux 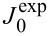 ≈ 3480 nm^−2^ms^−1^. Following the earlier studies of FEBID using IDMD [13,15] the dwell time value 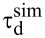 = 10 ns is used in the simulations. The rescaled electron current in the simulations is thus *I*_sim_ ≈ 4.375 μA.

#### Replenishment

During the replenishment phase precursor molecules are not exposed to the electron beam, while volatile fragments leave the surface and new precursor molecules are adsorbed. The characteristic time of the replenishment phase in FEBID experiments is of the order of milliseconds [5]. The amount of the added precursor molecules in combination with the electron current density defines whether the FEBID process runs in the electron-limited regime or the molecule-limited regime [6]. The electron-limited regime corresponds to a large number of precursor molecules with a small number of electrons leading to slow fragmentation and accumulation of the residual precursors. In the case of the molecule-limited regime, the number of secondary electrons exceeds the number of adsorbed precursors, causing dissociation of a larger number of molecules.

The realistic experimental time scale for the replenishment phase is challenging for MD simulations as it would require simulating the slow adsorption and desorption of new precursors and the created molecular fragments from the surface occurring on relatively large time scales. For specific values of pressure and temperature, the duration of the replenishment phase defines the concentration of adsorbates and the amount of desorbed molecules at the beginning of the next irradiation phase. If the replenishment time is long enough, the steady state is achieved. For shorter replenishment times, the concentration of adsorbates will be a fraction of the steady-state concentration.

Similarly to the creation of the initial precursor layer (see step 3), the replenishment phase is simulated to reproduce the physical state of the system after the replenishment, which is characterized by a certain number of desorbed fragments and the spatial distribution of newly adsorbed precursor molecules. The amount and spatial distribution of the precursor molecules added at each replenishment step can be varied in the model to describe different experimental conditions. The spatial distribution of the adsorbates added within the replenishment phase can also be modified depending on the injection method of precursor gas. The weakly bound precursor molecules and fragments are removed from the system by an artificial external force field. This procedure can be adjusted to the experimental desorption rate. After that, a new layer of precursors is created at a certain distance above the surface, optimized and deposited upon the substrate. Thermalization and relaxation of the system are performed at the end of the replenishment stage, which is then followed by the next cycle of irradiation.

In the present study each replenishment phase is simulated for 0.4 ns. New precursor molecules are deposited over the area of 14 nm × 14 nm to cover the beam spot while preventing the accumulation of the non-fragmented molecules along the perimeter of the simulation box where the fragmentation probability is very low (see Figure 3B). The density of new molecular layers is adjusted to maintain a balance between the number of added molecules and the number of fragmented molecules during the irradiation step. The surface density of the added Pt(PF_3_)_4_ layer is 0.3 and 1.2 molecules per nm^2^ for *E*_dep_ = 205 kcal/mol and *E*_dep_ = 300 kcal/mol, respectively. The deposition and thermalization simulations for each new layer are performed in the same way as for the initial precursor layer.

### Step 5. Post-deposition processing

Additional processing of the FEBID-grown structures, namely post-deposition electron irradiation or high-temperature annealing with or without additional gas (e.g., O_2_, H_2_O) [52–53], is commonly employed to purify the deposited material. According to the experimental data [54–55] as-grown FEBID structures usually contain a relatively low (ca. 10–30%) amount of metal, whereas post-irradiation processing of the deposits enables to significantly increase the metal content up to 80%. Energy transferred into the system by heating or via the electron–molecule interaction activates the desorption of volatile molecules from the surface and leads to rearrangement of the deposited structures. As a result, residual precursor molecules and fragments are removed, and remaining metal deposits are reorganized into more compact and dense structures.

Both types of post-deposition processing, that is, annealing and further electron irradiation, can be simulated by means of MD as the last step of the computational protocol shown in Figure 1. MD simulation of the annealing includes heating of the deposited structures to high temperature and subsequent slow cooling. During annealing structural and topological changes in the deposit take place. The typical time scale for annealing in experiments varies from minutes to several hours [55–57]. Therefore the temperature and the duration of the annealing in the simulations should be rescaled to match the amount of heat transferred to the deposited structures in experiments.

The simulation of the post-growth electron irradiation without depositing new precursors should be performed in a similar way as step 4, but without the replenishment phase. This process is characterized by the space- and energy-resolved distribution of the electrons, the electron flux, and the duration of the irradiation. If the deposited structure is relatively thin (e.g., of sub-monolayer thickness), as a first approximation one can use the same electron distribution as for the FEBID irradiation step. Otherwise, the SE distribution should be recalculated to account for the electron transport in both the substrate and the deposited material.

The computational procedure for atomistic modeling of the post-deposition effects has been included in the presented methodology shown in Figure 1 for the sake of completeness. Several test simulations of post-annealing and irradiation have been performed to verify the method, but a systematic analysis of these effects goes beyond the scope of the present paper and will be a subject of a separate study.

## Results and Discussion

The protocol described above for atomistic IDMD simulations of the FEBID process using the MBN Explorer and MBN Studio software has been used to simulate the formation of Pt-containing nanostructures during the FEBID of Pt(PF_3_)_4_ molecules. The results of these simulations are described in this section. In particular, the detailed structural analysis of nucleated metal clusters is in focus.

**Figure 4:**
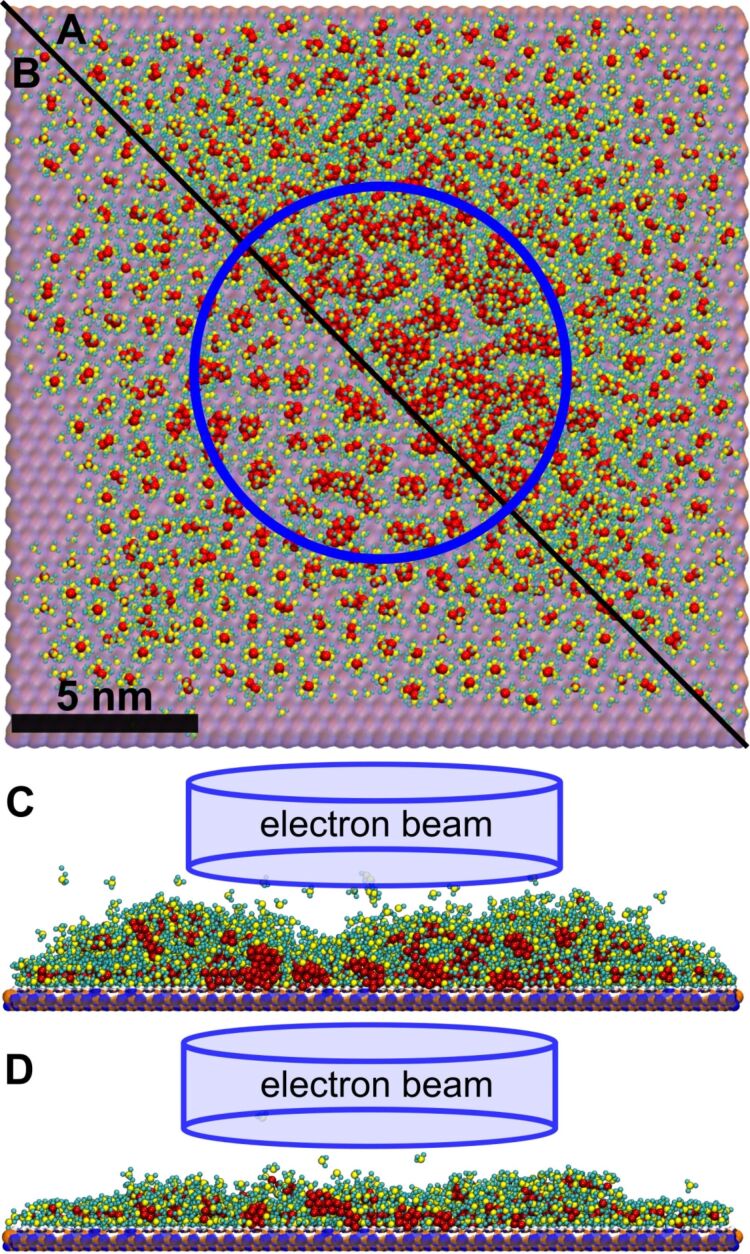
A snapshot of the multiscale IDMD simulation of a FEBID process using Pt(PF_3_)_4_ at the end of the 20th cycle (after 200 ns of the simulation). Panels (A) and (C) show top view and side view of a 6 nm thick slice through the beam center of the grown structure for an energy transferred to the medium during fragmentation of *E*_dep_ = 300 kcal/mol with 223 precursor molecules added on average per FEBID cycle. Panels (B) and (D) show the corresponding views for the regime of *E*_dep_ = 205 kcal/mol with 68 precursor molecules added on average per FEBID cycle. The scale bar is the same for all figures. The blue cylinder indicates the PE beam spot. The images have been rendered using “Visual molecular dynamics” software [58].

IDMD simulations have been performed for 30 cycles of the FEBID process with a total simulation time of 300 ns. The accumulated fluence of PE is ca. 1.04 × 10^19^ cm^−2^, which corresponds to the equivalent total experimental irradiation time of 30 ms with the beam parameters from [22]. A snapshot of the system at the end of the 20th FEBID cycle is presented in Figure 4. Panels A–D of Figure 4 correspond to different amounts of energy transferred to the medium via Pt–P bond fragmentation. The energy parameter *E*_dep_ governing the bond fragmentation is equal to 300 kcal/mol and 205 kcal/mol for the cases presented in panels (A, C) and (B, D), correspondingly. As shown in Figure 4A and Figure 4B, three spatial regions can be distinguished where different structures are formed on the surface depending on the spatial distributions of fragmentation probability and adsorbed precursor molecules. Inside the beam spot area with a diameter of 10 nm (indicated by the blue circle in Figure 4A and Figure 4B) the high probability of Pt–P bond fragmentation leads to dissociation of Pt(PF_3_)_4_ molecules and the formation of metal clusters. The clusters grow, merge, and interconnect during the irradiation process, forming a network of thread-like metallic nanostructures. The transition region of 1 nm radius outside the beam spot area contains smaller metal clusters with a larger number of PF_3_ ligands attached due to lower fragmentation probability in this region. The presence of the ligands prevents dense packing and aggregation of isolated metal clusters. As a result, the height of the deposited structures in this region is higher than that within the beam spot area. The region beyond the transition region (near the simulation box boundaries) contains mostly intact or less fragmented precursor molecules.

The growth of Pt-containing clusters occurs faster with an increase of *E*_dep_. Within the *E*_dep_ energy range considered, the fragmentation of Pt(PF_3_)_4_ molecules is the most prominent at *E*_dep_ = 300 kcal/mol (see Figure 4A and Figure 4C). This can be explained by a lower probability of rejoining the broken bonds at the higher amount of energy deposited. The number of precursor molecules added at each FEBID cycle is approximately equal to the number of fragmented molecules. The average number of added molecules is equal to 68 and 223 for *E*_dep_ = 205 kcal/mol and *E*_dep_ = 300 kcal/mol, respectively. These numbers correspond to a surface density of 0.3 and 1.2 molecules/nm^2^, respectively. The larger amount of precursors adsorbed on the surface in the FEBID process leads to a faster accumulation of Pt atoms enabling the formation of larger metal clusters.

**Figure 5:**
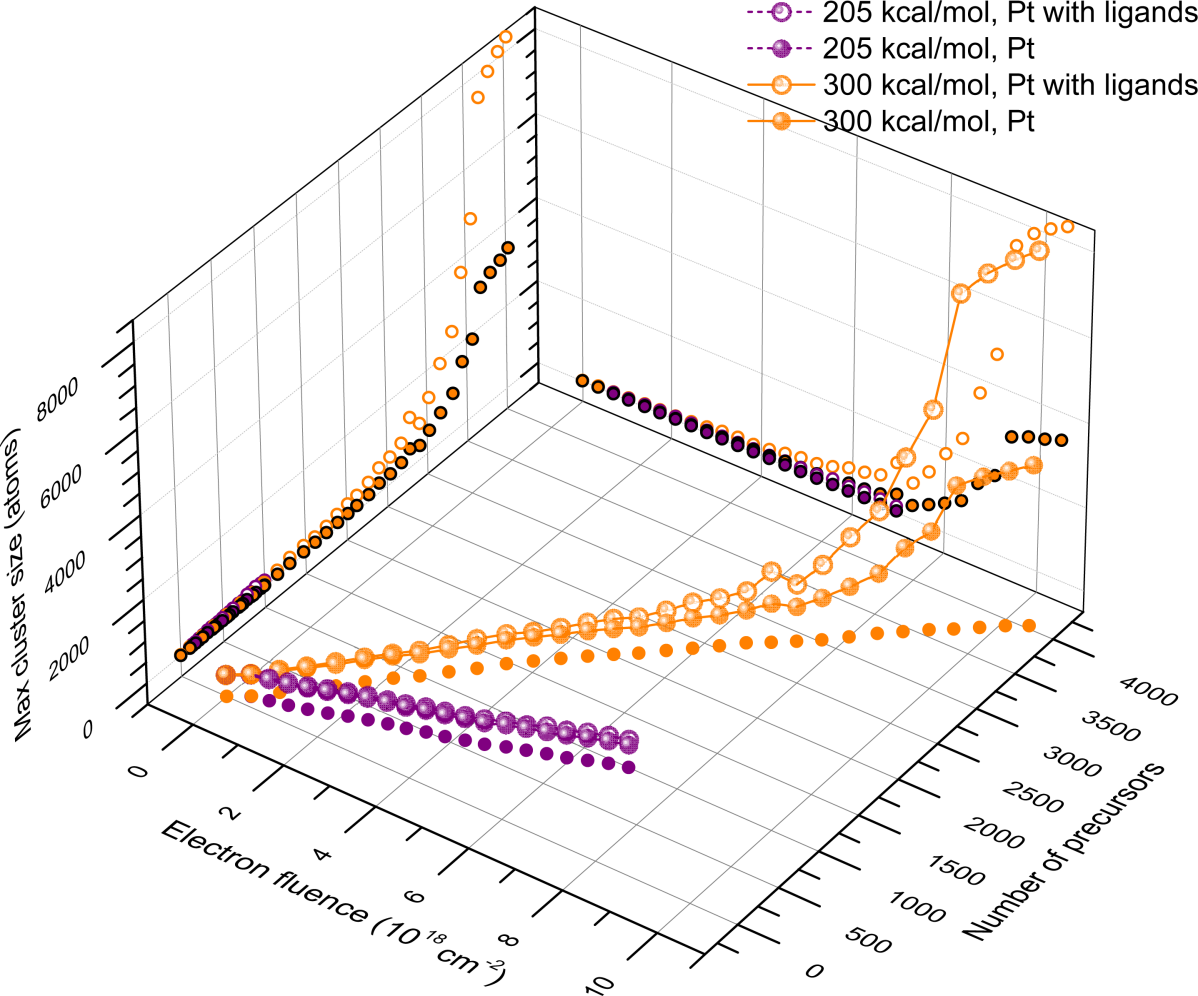
The number of Pt atoms (full spheres) and the total number of atoms including ligands (open spheres) in the largest cluster as functions of electron fluence and the number of adsorbed precursor molecules in the effective beam spot for energies transferred to the medium during fragmentation of *E*_dep_ = 205 kcal/mol (purple symbols) and 300 kcal/mol (orange symbols). Full and open dots show the respective projections on the planes. Each symbol describes the parameter values derived at the end of each consecutive FEBID cycle.

The growth of the deposits can be quantified by the total number of atoms and the number of metal atoms in the largest cluster. Figure 5 shows the evolution of maximal cluster size for *E*_dep_ = 205 and 300 kcal/mol as a function of electron fluence and the number of Pt(PF_3_)_4_ molecules deposited within the beam spot area (with a radius of 5 nm) surrounded by a diffusive halo with a width of 1 nm. This spatial region (denoted hereafter as the effective beam spot area) is characterized by the highest fragmentation probability (see Figure 3B) due to secondary and backscattered electrons. Figure 5 illustrates that several stages of nanostructure growth can be distinguished. For the case of *E*_dep_ = 300 kcal/mol, fragmentation of the precursor molecules leads to the formation of isolated small clusters during the first 5–6 FEBID cycles (corresponding to an electron fluence of ca. 2 × 10^18^ cm^−2^). This regime is characterized by a linear dependence of the largest structure size on electron fluence (see orange dots in Figure 5). During further irradiation the clusters start to merge while the structure growth continues simultaneously due to the deposition of new precursor molecules. An interplay of these phenomena results in a much faster increase of the number of atoms in the largest cluster as a function of electron fluence. The growth of Pt clusters as a function of the number of added precursor molecules follows the same trend for both values of *E*_dep_, but the growth rate is higher at the larger *E*_dep_. Simultaneously, a much faster growth rate as a function of electron fluence is observed for *E*_dep_ = 300 kcal/mol. The similar evolution of the maximum cluster size for both values of *E*_dep_ suggests that the results obtained in the two studied regimes can be scaled.

The maximum size of the metal cluster, *N*_max_, is determined by the number of metal atoms located in the effective beam spot area. The latter number depends, in turn, on the number of precursor molecules, *N*_p_, adsorbed in that spatial region under given irradiation conditions. The dependence of *N*_max_ on *N*_p_ follows the empirical dependence represented by the two terms corresponding to the two regimes of cluster growth described above:

[8] $$N_{\text{max}}=aN_{\text{p}}+N_{\text{lim}}{\left(1+e^{-\frac{N_{\text{p}}-N_{\text{tr}}}{\Delta N}}\right)}^{-1}.$$

Here *a* is the dimensionless coefficient of the initial linear growth of clusters. The beam spot size limits the maximal transversal size of the formed metal nanostructure. This condition is accounted for by introducing a sigmoid function, the second term on the right-hand side of Equation 8. Δ*N* is an interval of *N*_p_ values within which the morphological transition from isolated islands to a single nanostructure takes place. The parameter *N*_lim_ stands for the limiting cluster size at which the morphological transition will be completed. *N*_tr_ is the sigmoid’s midpoint that defines the number of adsorbed precursor molecules enabling the morphological transition. In general, all these parameters depend on the irradiation and the replenishment conditions as well as on the value of *E*_dep_.

**Figure 6:**
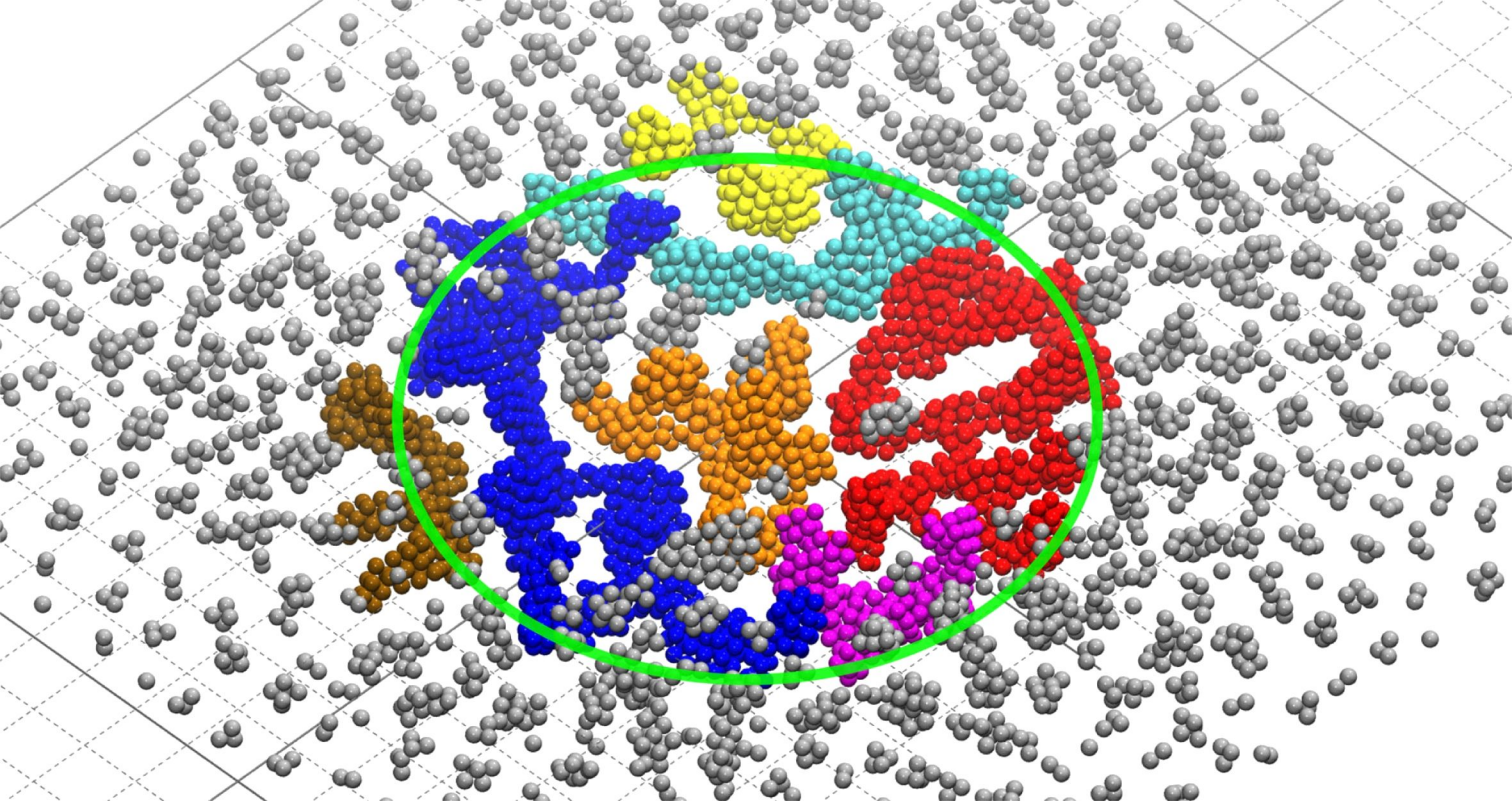
A snapshot of the IDMD simulation of the FEBID process of Pt(PF_3_)_4_ in the regime of *E*_dep_ = 300 kcal/mol at an electron fluence of 8 × 10^18^ cm^−2^. Only Pt atoms are shown. Separate clusters of a size larger than 150 atoms are shown in different colors, while smaller clusters are shown in grey. The green circle indicates the PE beam spot. The grid spacing is 1 nm.

Equation 8 permits the quantitative description of the morphological transition from separate clusters into a single structure. As shown previously [13], when isolated metal islands merge into a single nanostructure, its further growth occurs due to the attachment of fragmented precursor molecules to it. In this regime the maximal size of the metal structure follows a linear dependence on electron fluence and the number of precursor molecules. The parameters of the sigmoid function can be defined with high precision at least after passing the transition point. As shown in Figure 6, several large clusters containing more than 150 Pt atoms have been formed in the beam spot area in the present simulation at electron fluence of 8 × 10^18^ cm^−2^. Within the next several cycles these clusters merge together and the structure starts to grow by the attachment of new material to the existing deposit. This effect manifests itself as a change of the growth rate during the last four simulation cycles in Figure 5.

The number of precursor molecules added at each FEBID cycle depends on the value of *E*_dep_. As seen from Figure 5, the dependence of *N*_p_ on the electron fluence *F* can be approximated by a linear function:

[9] $$N_{\text{p}}=k\left(E_{\text{dep}}\right)\cdot S_{0}\cdot F\;,$$

where *S*_0_ = 1.1 × 10^−16^ cm^2^ is the area of the spherical region with a radius of 6 nm. Equation 9 is valid for the case when the amount of adsorbates added during each sequential FEBID cycle is kept nearly constant. In the present case, this amount is defined by the number of fragmented precursors. The ratio of newly delivered and fragmented precursors defines whether the considered regime is precursor-limited or electron-limited. Fitting the data plotted in Figure 5 yields the values *k*_205_ = 1.3 × 10^−4^ and *k*_300_ = 3.6 × 10^−4^ for *E*_dep_ = 205 and 300 kcal/mol, respectively. Substituting Equation 9 in Equation 8, one derives the dependence of the maximal cluster size on the electron fluence:

[10] $$N_{\text{max}}=ak\left(E_{\text{dep}}\right)S_{0}F+N_{\text{lim}}{\left(1+e^{-\frac{kS_{0}F-N_{\text{tr}}}{\Delta N}}\right)}^{-1}.$$

Equation 10 has been used to fit the simulated dependencies *N*_max_(*F*) for *E*_dep_ = 205 and 300 kcal/mol with the variable parameters *a*, Δ*N*, *N*_tr_ and *N*_lim_, whereas the values of *k* have been fixed. As the dependence of *N*_max_ on *N*_p_ is practically the same at different *E*_dep_ (see Figure 5), the variable parameters are set the same for both dependencies. The fitting procedure yields the values summarized in Table 3. The successfully converged fit confirms that the variable parameters defining the growth rate of the clusters do not depend on *E*_dep_.

**Table 3:** Parameters from Equation 10 providing the best fit of the analytical dependence with the one obtained from simulations. *k* is a scaling coefficient of the number of adsorbed precursors, *S*_0_ is the effective beam spot area, *N*_lim_ is the estimated limiting number of Pt atoms in the nanostructure, *N*_tr_ is the number of adsorbed precursors in the transition point, Δ*N* is an interval of *N*_p_ values within which the morphological transition takes place, and *a* is the coefficient of the linear cluster growth.

Parameter	*E*_dep_	
	205 kcal/mol	300 kcal/mol

*k*	1.3 × 10^−4^	3.6 × 10^−4^
*S*_0_, cm^2^	1.1 × 10^−16^	
*N*_lim_	3174	
*N*_tr_	3544	
Δ*N*	181	
*a*	8.1 × 10^−2^	

The similar *N*_max_(*N*_p_) dependence at different *E*_dep_ values permits to explore computationally this dependence at larger *E*_dep_ (as such simulations are significantly faster) and then rescale it to the physically meaningful, smaller value of *E*_dep_ and larger fluence *F* according to Equation 9. One should note that similar *N*_max_(*N*_p_) dependencies have been observed when the number of precursor molecules added during each FEBID cycle is approximately equal to the number of fragmented molecules for both values of *E*_dep_. The possibility of scaling for different FEBID regimes will be explored in future studies.

In the following subsections the deposited nanostructures are characterized in greater detail by considering the evolution of (i) the individual cluster size and shape, and (ii) height and metal content of the whole deposited structure in the beam spot area as a function of electron fluence or the number of irradiation cycles. The results presented below correspond to the case of *E*_dep_ = 300 kcal/mol.

### Characterization of individual clusters

**Figure 7:**
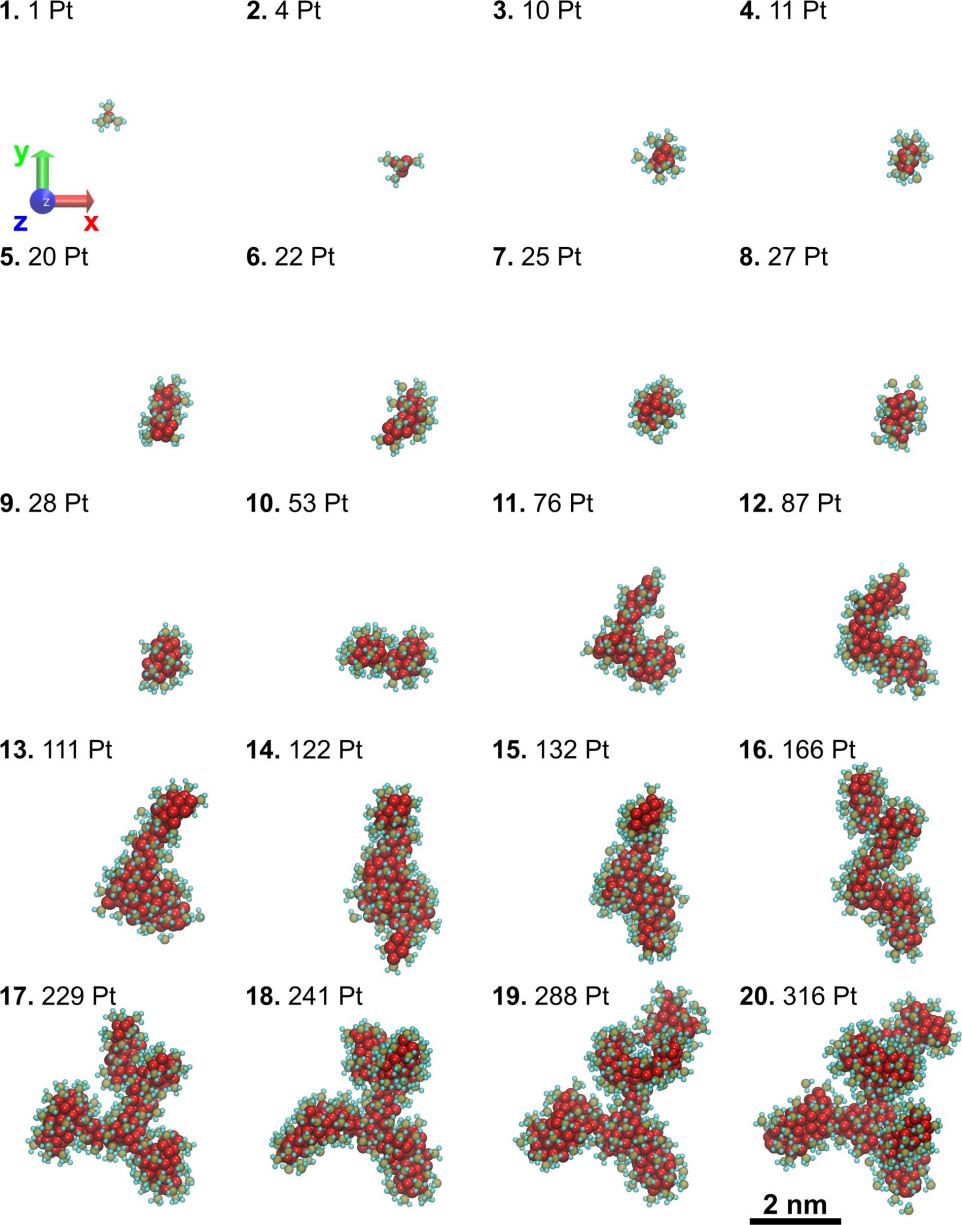
Evolution of the largest cluster in the course of simulation at the end of each FEBID simulation cycle (shown in bold). The number of Pt atoms is also indicated for each structure.

One of the largest clusters formed in the course of the simulation has been selected to study its evolution during the FEBID process. The evolution of the cluster is tracked back to the initial nucleation stage using coordinates of the center of mass of the metal core. The cluster structure and the number of Pt atoms in the cluster at the end of each FEBID cycle are shown in Figure 7. The platinum-containing nanostructure grows via coalescence of the neighboring metal clusters of different sizes. The coalescence takes place via an interplay of the following mechanisms: (i) an addition of a fragmented precursor molecule with a single Pt atom (see the evolution of the cluster structure at FEBID cycles 3–4 and 8–9), (ii) the merging of two clusters of comparable sizes (see the cluster structure at the cycles 4–5, 9–10, and 10–11), as well as a combination of both. Metal clusters containing up to about 30 Pt atoms preserve a spherical shape and tend to rearrange after an elongation caused by the merging of clusters of comparable size (see cycles 1–9). The sequential coalescence of larger clusters containing several tens of Pt atoms results in the formation of randomly oriented branched structures (see cycles 10–20). The continuation of the irradiation and replenishment processes leads to further growth and interconnection of the branched clusters into a single metal network observed in this simulation at the 27th cycle corresponding to a PE fluence of 9.4 × 10^18^ cm^−2^ (see Figure 5).

**Figure 8:**
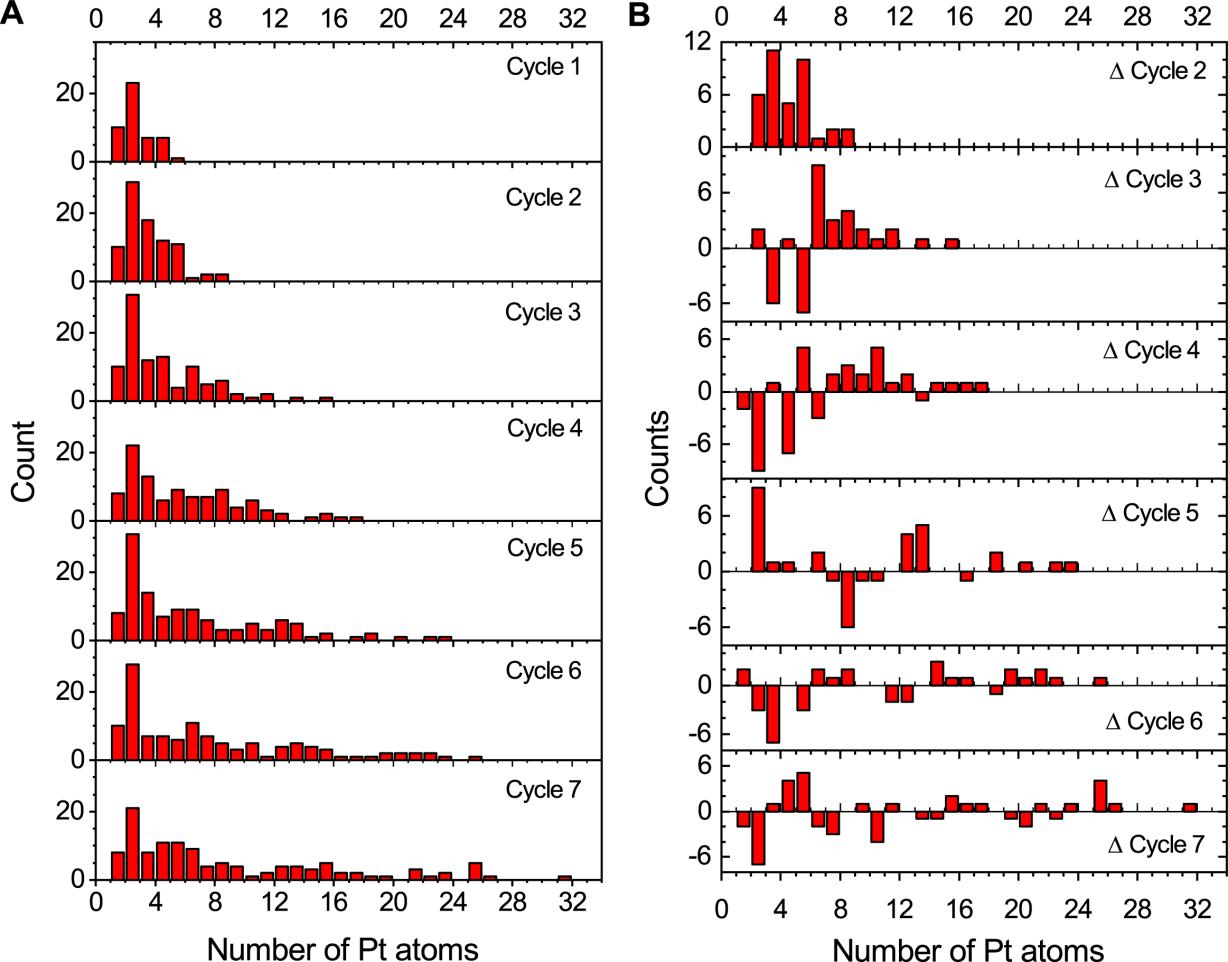
(A) Size distribution of Pt clusters during the first seven FEBID cycles. (B) Difference of size distributions for Pt clusters between two consecutive FEBID cycles for the first seven cycles.

Next, the process of initial cluster growth and coalescence has been studied for an ensemble of the deposited clusters. Figure 8 shows the size distribution of grown clusters as a function of the number of Pt atoms (Figure 8A) and the difference between the distributions for two consecutive FEBID cycles (Figure 8B) for the first seven FEBID cycles. In the course of these cycles small metal clusters start to nucleate and reach a size of about 20–30 atoms. As demonstrated in Figure 7, clusters of substantially larger size are formed during the several follow-up FEBID cycles when clusters of similar size, which have been formed over the first seven cycles, merge. The distributions shown in Figure 8 are obtained at the end of the irradiation stage at each FEBID cycle. Positive values in Figure 8B indicate an increased number of clusters of a given size in comparison with the previous cycle; negative values indicate a decreased number of the clusters of such size. As more irradiation cycles are performed and more Pt atoms are accumulated on the surface due to replenished precursors, the Pt-containing structures start to merge and consistently increase in size. The size distributions shown in Figure 8A peak at a number of platinum atoms *N* = 2 for all the FEBID cycles considered. This is attributed to the constant addition of precursor molecules during the replenishment stage at each FEBID cycle. During the following cycles Pt-containing structures are formed mainly via coalescence of larger clusters containing about 20–30 Pt atoms. When the clusters reach a certain size, they become less mobile and behave as centers of attraction for new molecules.

The morphology of the formed metal clusters has been characterized by analyzing their fractal dimension. The fractal dimension *D* of a cluster is calculated by evaluating its center of mass and measuring the number of atoms contained inside the circumscribed sphere of radius *R* [59]. The number of atoms *N* scales with the radius *R* as

[11] $$N\left(R\right)\propto R^{D},$$

or equally

**Figure 9:**
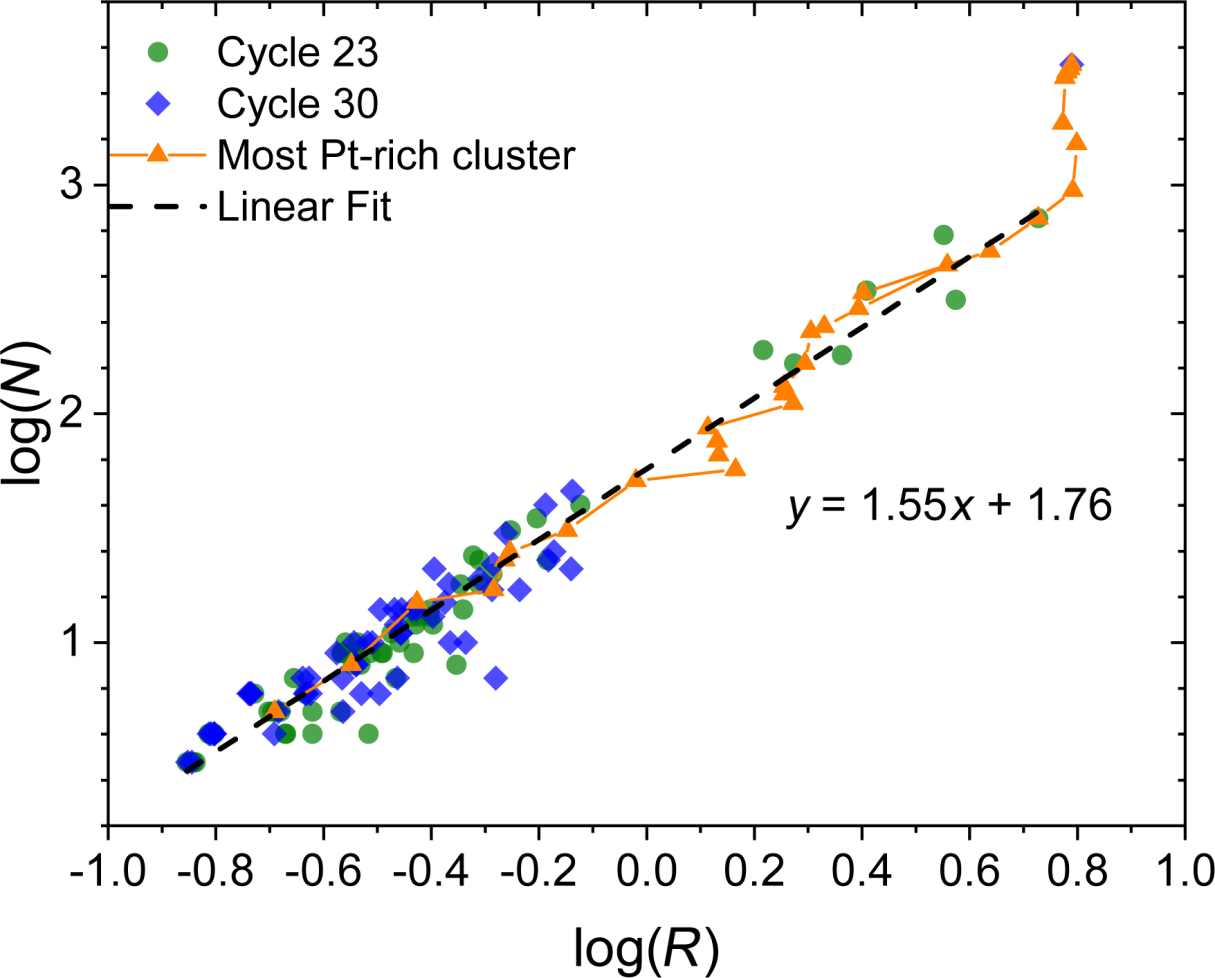
Double-log plot of the number of Pt atoms *N* in the deposited clusters as function of the radius *R* of the circumscribed sphere. Green dots and blue diamonds correspond to the whole ensemble of deposits at the end of the 23rd and 30th simulation cycle, respectively. Interconnected orange triangles describe the cycle-by-cycle growth of the most Pt-rich clusters. The dashed line shows a linear fit of the log(*N*)-vs-log(*R*) distribution of the clusters at the end of the 23rd simulation cycle.

[12] log(N)=D⋅log(R)+C,

where *C* is a constant. Equation 12 enables one to determine the fractal dimension *D* by considering the double-log dependence of the number of atoms in a cluster on its circumscribed sphere radius. Results of this analysis performed for the cycle-by-cycle evolution of the most Pt-rich clusters are shown in Figure 9 by interconnected orange triangles. The radius of the circumscribed sphere for the largest Pt cluster stops growing after the 24th cycle when the value of *R* = 6.2 nm corresponding to the radius of the effective beam spot is reached. Green dots in Figure 9 show the fractal dimension evaluated for all the metal clusters in the beam spot area at the 23rd cycle prior to the merging of separate clusters into a single structure. Blue diamonds show the corresponding values of *D* for all the metal clusters in the beam spot area at the 30th (final) simulation cycle.

Overlapping of the values for the single cluster at different FEBID cycles and for the whole ensemble of clusters for the last cycle indicates the co-existence of clusters at various growth stages. The calculated log(*N*) values exhibit a linear dependence on log(*R*) with the fractal dimension *D* = 1.55 and the constant *C* = 1.76. The evaluated value of *D* lies between the values *D*_1D_ = 1 and *D*_2D_ = 2 corresponding to ideal linear and planar structures, respectively, and it is within the typical range of values known for fractal-like metal clusters and aggregates [60–61].

**Figure 10:**
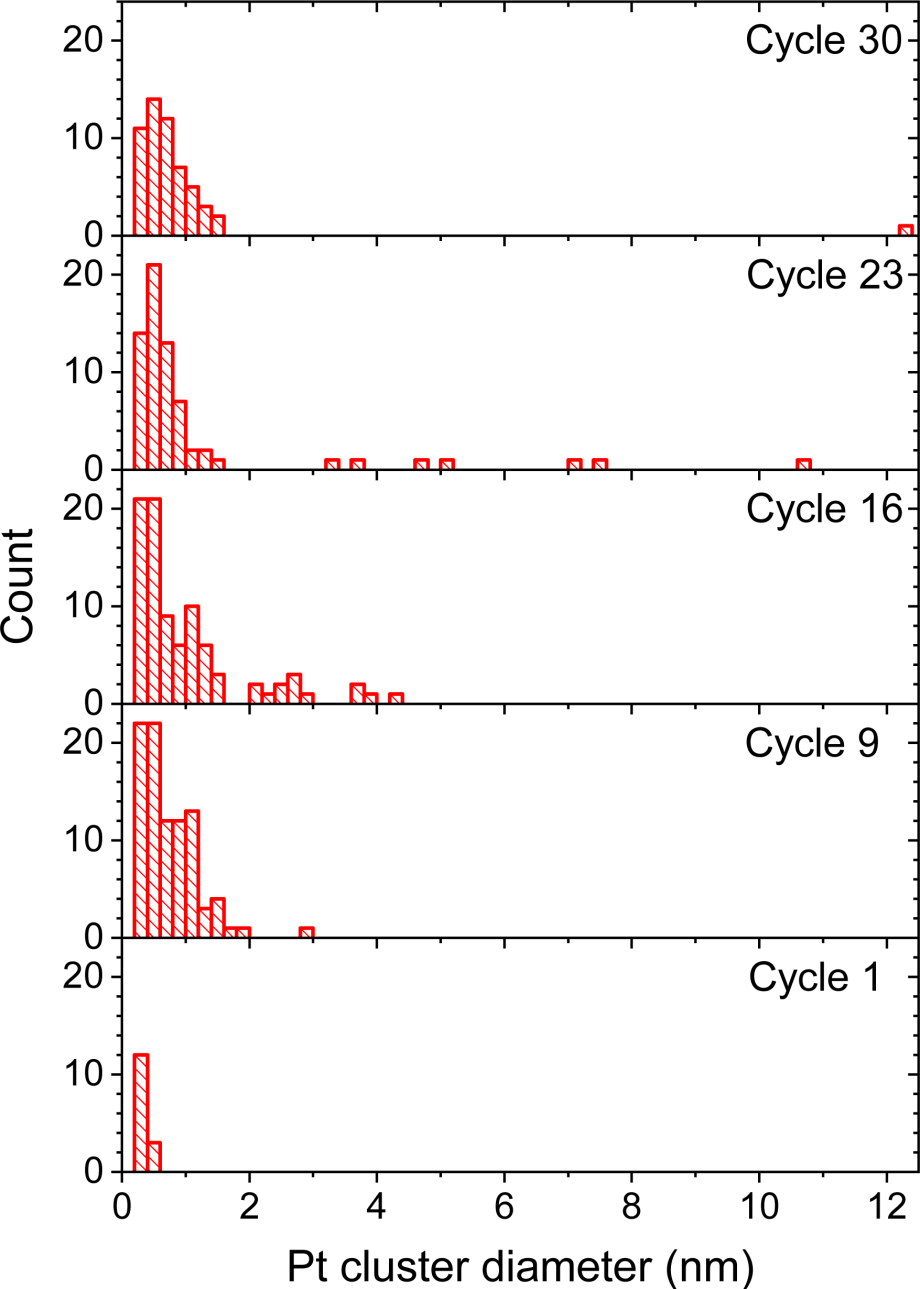
Distribution of the circumvented diameters *d* of the deposited Pt clusters after several cycles of the FEBID simulation for *E*_dep_ = 300 kcal/mol.

The distribution of circumvented diameters *d* of the deposited clusters at different cycles is shown in Figure 10. Only clusters larger than dimers are included in this analysis. A cluster containing approx. 20 Pt atoms is circumscribed by a sphere of a diameter of around 1 nm. Under the conditions considered in this study, separate metal clusters merge into dendritic structures. The consecutive growth of the cluster diameter is observed until the instant when most deposited atoms merge into a single nanostructure. At this stage the nanostructure covers an effective round beam spot area with a diameter of approx. 12 nm. Further growth of the cluster will occur via an increase in the nanostructure height.

### Evaluation of nanostructure height and its metal content

Metal content and height of the nanostructures are the main experimentally measured FEBID characteristics. The experimental results [21] indicate that the growth rate and atomic content of the deposited material strongly depend on the electron fluence and the amount of adsorbed precursor molecules.

As shown in Figure 4, the metal clusters in the simulations grow nearly isotropically in the (*xy*)-plane parallel to the substrate surface, but the cluster size and morphology depend on the radial distance from the beam center. Thus, the height and the relative Pt content of the deposited nanostructures are evaluated in concentric bins with a width of 1 nm around the beam spot axis.

**Figure 11:**
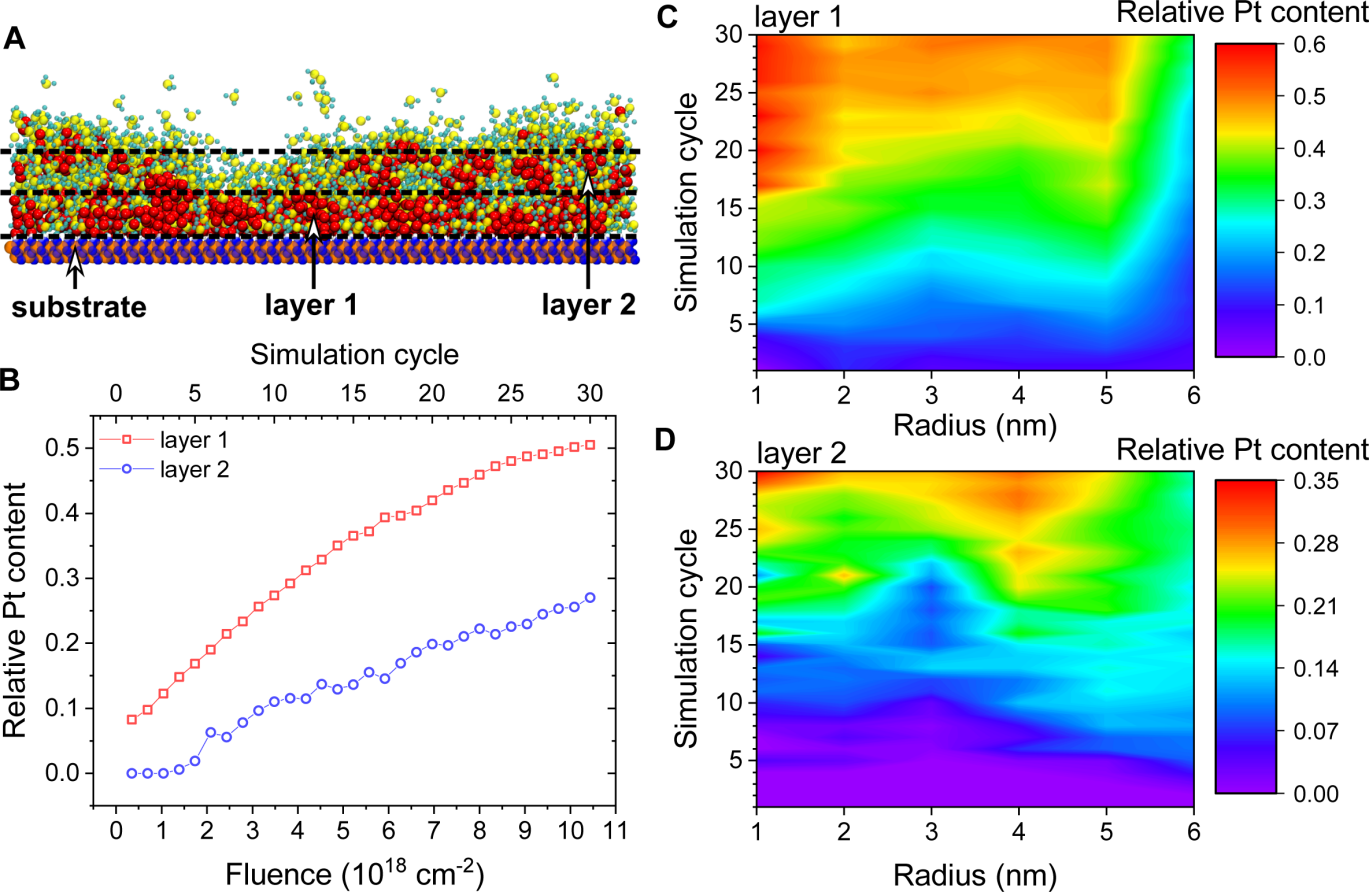
(A) The atomic content of the deposited nanostructure is analyzed by splitting the nanostructure into layers of 0.7 nm thickness each, corresponding to the height of a Pt(PF_3_)_4_ monolayer. (B) Relative Pt content in the beam spot area for the first two layers of 0.7 nm thickness as a function of the electron fluence. Panels (C) and (D) show the evolution of the radial distribution of relative Pt content for the first and second layers, respectively.

The nanostructure growth during the FEBID process is characterized by the atomic content of the deposited material. The relative atomic content is calculated considering all atoms in the beam spot area layer by layer. The thickness of each layer is set to 0.7 nm corresponding to the height of a Pt(PF_3_)_4_ monolayer adsorbed on SiO_2_ (see Figure 11A). The relative Pt content is calculated by dividing the number of Pt atoms by the total number of atoms in the considered volume. The evolution of the average Pt content in the first two layers with the number of FEBID cycles is presented in Figure 11B–D. Figure 11B shows the relative Pt content as a function of the electron fluence. Figure 11C and Figure 11D show the evolution of the radial distribution of Pt atoms for the first two layers.

Figure 11B reveals that the relative Pt content in the first layer increases linearly during the first eleven FEBID cycles and then the growth slows down, eventually coming to saturation. This indicates that the formation of the first layer has been completed within the 30 FEBID cycles. Note that the first layer has undergone minor structural transformations even after the metal clusters merged into a single structure. Further evolution of the structure will concern mainly the second and above layers. The Pt distribution within the first layer is nearly homogeneous during the first 15 simulation cycles, while a more dense region proximal to the beam axis appears after the 15th cycle. This region corresponds to the location of the largest cluster in the simulation. Platinum atoms start filling the second layer at the 5th cycle at the edge of the beam spot.

**Figure 12:**
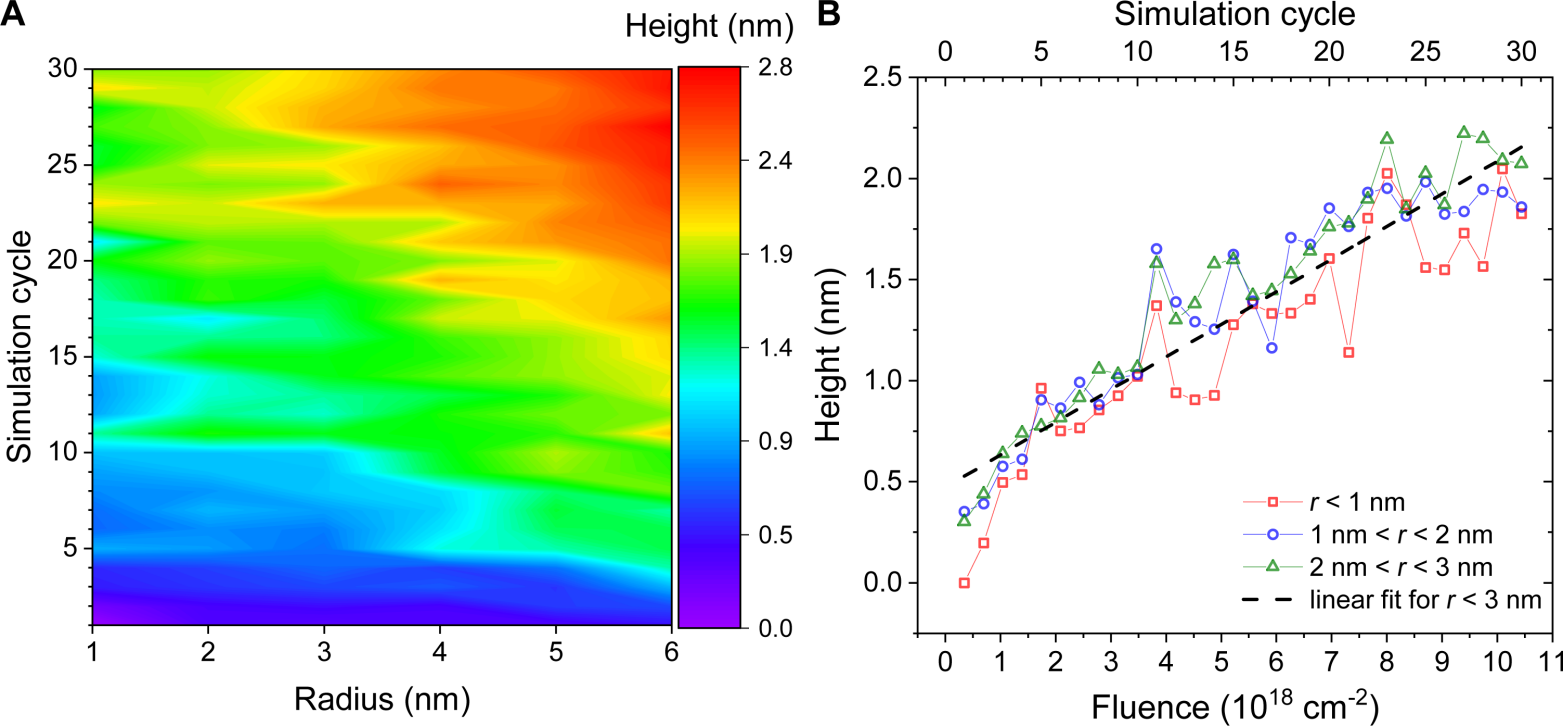
Evolution of the height of the grown Pt-containing structure at different FEBID simulation cycles for *E*_dep_ = 300 kcal/mol. (A) Maximum height of deposited Pt structures within concentric bins of 1 nm thickness from the electron beam axis at the end of each simulated FEBID cycle. (B) Maximum height of Pt structures within the distance of 3 nm from the electron beam axis as a function of electron fluence fitted by a linear function.

The thickness of the deposited material is calculated by the maximum *z*-coordinate of Pt atoms within the concentric bins. Figure 12A shows the evolution of the dependence of the nanostructure height as a function of the radius from the PE beam axis in the course of the FEBID simulation. Two regions can be distinguished, that is, one in the center of the beam spot and another one closer to its edge (see also Figure 11A). The larger height of the structures at distances of 4–6 nm from the beam center arises due to the presence of attached PF_3_ ligands, which do not permit dense packing of the Pt clusters. The height of the deposited material in the center of the beam spot (at radial distances up to 3 nm from the beam axis) as a function of the electron fluence is shown in Figure 12B. Considering the experimental electron flux [22] of 3.48 × 10^17^ cm^−2^·ms^−1^, the height dependence exhibits a linear behavior with a growth rate of 0.067 nm/ms. The growth rate calculated based on the data from [22] is equal to 0.003 nm/ms. One should note that the two growth rates are evaluated at different regimes. The experimental growth rate is determined from the average linear growth of the 1758 nm high metal pillar irradiated with a stationary electron beam for 10 min. In the present simulations, the metal structure within the bottom-most atomic layer still undergoes minor structural transformations involving the reorganization of the metal clusters. After completing the first layer, the nanostructure growth should continue in a different regime with the growth rate being comparable to the experimental value. The difference between the experimental value of the growth rate and the value derived from the simulations can also be ascribed to different rates of the precursor deposition and the larger value of *E*_dep_.

## Conclusion

This study has presented a general protocol for the systematic atomistic modeling of the FEBID using the MBN Explorer and MBN Studio software packages. The protocol is based on the methodology developed previously [13,15] and is applicable to any combination of precursor, substrate, and electron beam parameters. Using the protocol, the irradiation-driven molecular dynamics (IDMD) simulations of the nanostructure growth can be performed for various irradiation and replenishment regimes corresponding to realistic experimental conditions.

The proposed computational methodology is applicable to characteristic system sizes and time scales that can be treated by the classical molecular dynamics approach. The model requires the specification of the following input data: interatomic potentials describing the interaction of atoms in the system, precursor fragmentation cross sections, and distributions of primary, secondary, and backscattered electrons. The outcome of the FEBID process is governed by a balance of different processes, such as adsorption, desorption, diffusion, and fragmentation of the precursor molecules. The cumulative contribution of all these processes defines the regime of the FEBID process. The presented methodology reproduces the state of the molecular system before, during, and after irradiation, without explicit simulations of all the aforementioned processes. The model permits the inclusion of other phenomena such as chemisorption or electron-stimulated desorption. The presented method is under continuous development, so a more detailed and accurate description of the essential processes will be included in future studies. Finally, while being focused on the FEBID process using a pulsed electron beam, the methodology can be adjusted to simulate the nanostructure formation by other nanofabrication techniques using electron beams, such as direct-write electron beam lithography.

Atomistic simulations provide the complete characterization of the morphology and internal structure of metal deposits. An early stage of the FEBID process (nucleation, growth, and coalescence of the metal clusters) involves the initial formation of the deposited layer, which drives the further growth of the material. Atomistic analysis of the simulation results provides spatially resolved relative metal content, height, and growth rate of the deposits, and the linear size of the clusters, which represent valuable reference data for the experimental characterization of the FEBID material.

The presented simulation workflow has been successfully utilized to analyze the nanostructure formation with Pt(PF_3_)_4_ precursor molecules. The performed simulation of 30 FEBID cycles corresponds to an early stage of the nanostructure formation and the creation of several metal-enriched layers. At this stage small metal-containing clusters start to grow, merge, and interconnect, forming branched structures. The process of metal atom nucleation and the formation of a single metal structure can be correlated with time-dependent electrical conductivity measurements during FEBID [62–63]. Coalescence of smaller metal clusters into a single structure should correspond to a jump in the electrical current through the deposit. The calculated fractal dimension of the metal structures is equal to 1.55, which is a typical value for nanostructures created by metal cluster deposition. The metal structure formed after merging separate clusters covers the effective beam spot area with a diameter of 12 nm. The average Pt content in the beam spot area is approximately 30%. These findings can be verified experimentally through STM analysis.
